# Cellular and molecular characterization of a stem rust resistance locus on wheat chromosome 7AL

**DOI:** 10.1186/s13104-016-2320-z

**Published:** 2016-12-07

**Authors:** Vincent Pujol, Jose Robles, Penghao Wang, Jen Taylor, Peng Zhang, Li Huang, Linda Tabe, Evans Lagudah

**Affiliations:** 1 CSIRO Agriculture and Food, GPO Box 1600, Canberra, ACT 2601 Australia; 2 Plant Breeding Institute, School of Life and Environmental Sciences, University of Sydney, Cobbitty, NSW 2570 Australia; 3 Department of Plant Sciences and Plant Pathology, Montana State University, Bozeman, MT 59717 USA

**Keywords:** Wheat, Stem rust, Ug99, 7AL resistance, 7DL suppressor, RNA-Seq

## Abstract

**Background:**

Wheat stem rust, caused by *Puccinia graminis* f. sp. *tritici*, is a major wheat disease which is mainly controlled through the release of resistant cultivars containing one or several resistance genes. Considerable effort has been put into the discovery of new resistance genes, but knowledge of their mechanisms of action is often lacking. In this study, the mechanism of resistance conferred by a recently discovered stem rust resistance locus on wheat chromosome 7AL was investigated through microscopic observations and RNA-sequencing, using the susceptible line Columbus and the independent, backcrossed, resistant lines Columbus-NS765 and Columbus-NS766.

**Results:**

Microscopic observations of infected leaves revealed that the resistance conferred by the 7AL resistance locus was initiated 2 days post-inoculation, upon the fungus entry into the plant through the stoma. Resistance was manifested by death of guard and epidermal cells adjacent to an infection site. Occasionally, similar observations were made in the susceptible line, suggesting that the resistance response was the same in all genotypes, but enhanced in the resistant lines. Transcriptomic analysis, combined with assignment of genes to wheat chromosomes, revealed a disproportionately high number of differentially expressed genes were located on chromosomes 7AL and 6A. A number of genes annotated as cysteine-rich receptor-like kinases were located on chromosome 7AL. Closer investigation indicated that the encoded proteins were in fact putative receptor-like cytoplasmic kinases. One of the putative RLCK genes contained a SNP marker previously shown to co-segregate with the 7AL resistance locus. The results also indicated the presence of a large introgression on chromosome 6A in both resistant lines, but whether it has any role in the resistance response is unclear.

**Conclusions:**

This study represents the first investigation on the resistance mechanism conferred by the wheat 7AL stem rust resistance locus. The resistance response was associated with pre-haustorial cell death, and the transcriptome analysis suggested putative receptor-like cytoplasmic kinases as candidate resistance genes for further investigation.

**Electronic supplementary material:**

The online version of this article (doi:10.1186/s13104-016-2320-z) contains supplementary material, which is available to authorized users.

## Background

Wheat stem rust, caused by the fungus *Puccinia graminis* f. sp. *tritici* (*Pgt*), is an important wheat and barley disease with a great destructive capacity [1]. Control of wheat stem rust mainly consists in the development of resistant cultivars through introgression of resistance genes, responsible for the pathogen recognition and the initiation of the resistance response. However, *Pgt* can quickly evolve and overcome these resistance genes. The Ug99 lineage, which is composed of highly virulent races that emerged in parts of East Africa and Middle East, is a perfect example. This lineage first overcame the long lasting resistance gene *Sr31*, then quickly became virulent to *Sr24* and *Sr36* [2–4]. As the Ug99 lineage remains a threat to food security and future outbreaks are expected [5], new resistance genes are continuously sought. These genes must be used wisely [6], and understanding the associated mechanism of resistance is essential for an efficient and durable protection against rapidly evolving *Pgt*.

In wheat, most resistance genes (R-genes) confer resistance against *Pgt* in a race-specific manner [7] and usually involve a hypersensitive response (HR), which is associated with the death of host cells [8]. This prevents biotrophic pathogens, such as rusts, from further colonizing the plant. HR is the typical defence response of the effector-triggered immunity (ETI), which is initiated through the direct or indirect recognition of pathogen proteins called effectors by specific plant R-proteins. As such, the race-specificity of R-genes is a consequence of the specific interaction between highly diverse effectors and R-proteins. This is opposed to the broad-range resistance conferred by the pattern-triggered immunity (PTI), which is induced through the recognition of microbial-associated molecular pattern (MAMPs) [8]. Contrary to effectors, MAMPs are strongly conserved between races and even species. Although plant immunity has been an intense field of study, many questions remain about the molecular mechanisms of resistance, notably in wheat.

We recently reported the discovery of a new stem rust resistance locus located on the long arm of the wheat (*Triticum aestivum* L.) chromosome 7A [9]. This 7AL resistance locus is effective against all *Pgt* races so far tested, including three members of the Ug99 group (TTKSK, TTKST and TTTSK) [9]. The 7AL locus was derived from wheat cultivar Canthatch (CTH), in which the resistance is not expressed. CTH is reported to contain a suppressor of stem rust resistance located on chromosome 7DL (7DL-Sup) [10–12], and we hypothesize that the 7AL resistance locus is in fact subject to suppression by the 7DL-Sup [9]. The 7AL resistance, and presumably other stem rust resistance specificities, were expressed in a number of independent EMS-generated CTH mutants in which the 7DL-Sup was reported to be inactivated [9, 11]. Two stem rust resistant lines (Col-NS765 and Col-NS766, BC_5_F_4_) were created by crossing two independent CTH non-suppressor (CTH-NS) mutants with the susceptible cultivar Columbus (Col), which hypothetically lacks both the 7AL resistance locus and the 7DL-Sup [9]. The 7AL resistance locus was mapped in a population (F_2:3_) derived from a cross between the resistant line Col-NS766 and the susceptible line Columbus. It was confirmed that the resistance locus was also present in Col-NS765, CTH-NS mutant lines and CTH [9].

The aim of this study was to characterize the resistance conferred by the 7AL locus at the cellular and molecular levels, using microscopy and RNA-sequencing (RNA-Seq) in the susceptible Columbus and resistant derivative lines. Moreover, the combination of mapping information and transcriptomics indicated possible candidates for the resistance gene(s) on 7AL.

## Results

### Stem rust resistance phenotyping

**Fig. 1:**
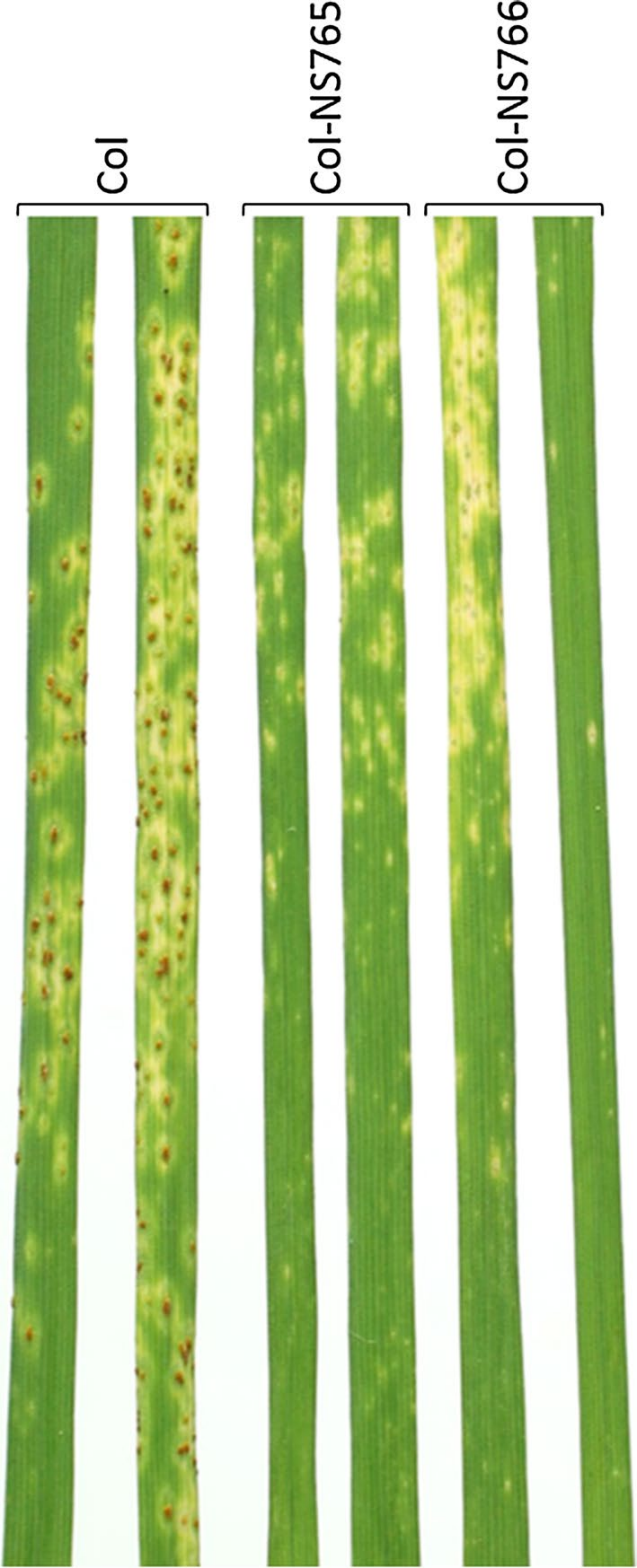
Resistance to *Pgt* culture no. 313 on seedlings, 11 DPI. Col was moderately susceptible (2^+^3), whereas Col-NS765 and Col-NS766 were resistant (;1)

Resistance against *Pgt* was assessed on seedlings, 11–15 days post-inoculation (DPI) with rust spores. Col was moderately susceptible (Infection type-IT 2^+^3), whereas Col-NS765 and Col-NS766 were resistant (IT ;1) (Fig. 1). CTH-K was fully susceptible (IT 3^+^4), whereas CTH-NS1 and CTH-NS2 were moderately resistant (IT 22^+^) (data not shown). Susceptible control Morocco, hardly having any known resistance genes for *Pgt*, was fully susceptible (IT 4) (data not shown). These results were in agreement with Pujol et al. [9].

### Microscopic observations of the stem rust resistance response

#### Fluorescein isothiocyanate wheat germ agglutinin staining

**Fig. 2:**
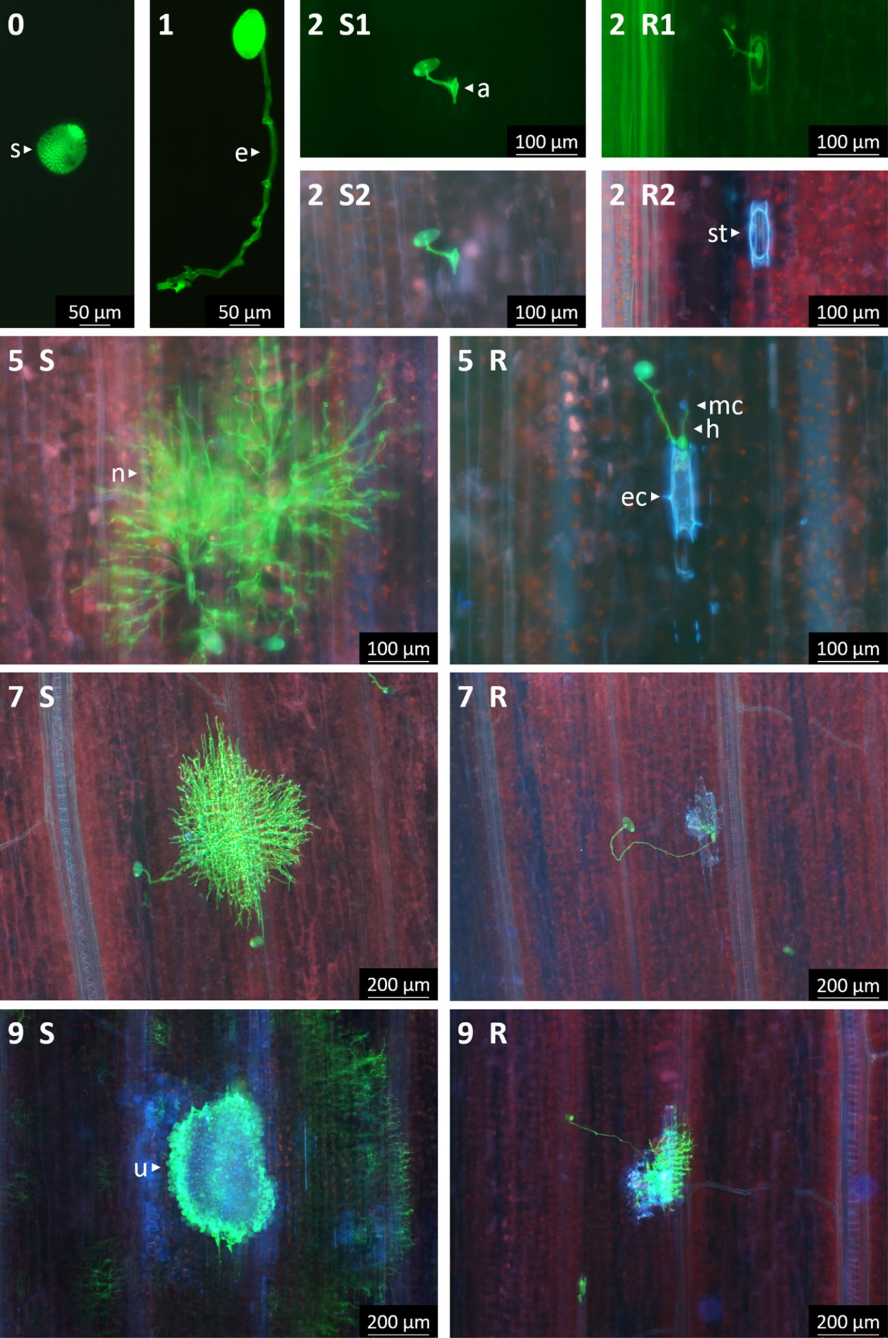
Rust development on resistant and susceptible genotypes. Histological observations of rust structures, stained with WGA-FITC, in Col, Col-NS765 and Col-NS766 throughout infection. Time points are indicated by the corresponding numbers (DPI), and *S* and *R* represent the typical responses of the susceptible and the two resistant genotypes respectively. *Arrows* indicate various rust and plant structures: urediniospore (s), elongation tube (e), appressorium (a), hypha (h), hyphal network (n), uredinia (u), stoma (st), epidermal cell (ec) and mesophyll cell (mc). *Pictures* 0, 1, 2S1 and 2R1 were taken under blue light, and the others under UV. 2S1/2S2 and 2R1/2R2 show the same field of view

**Fig. 3:**
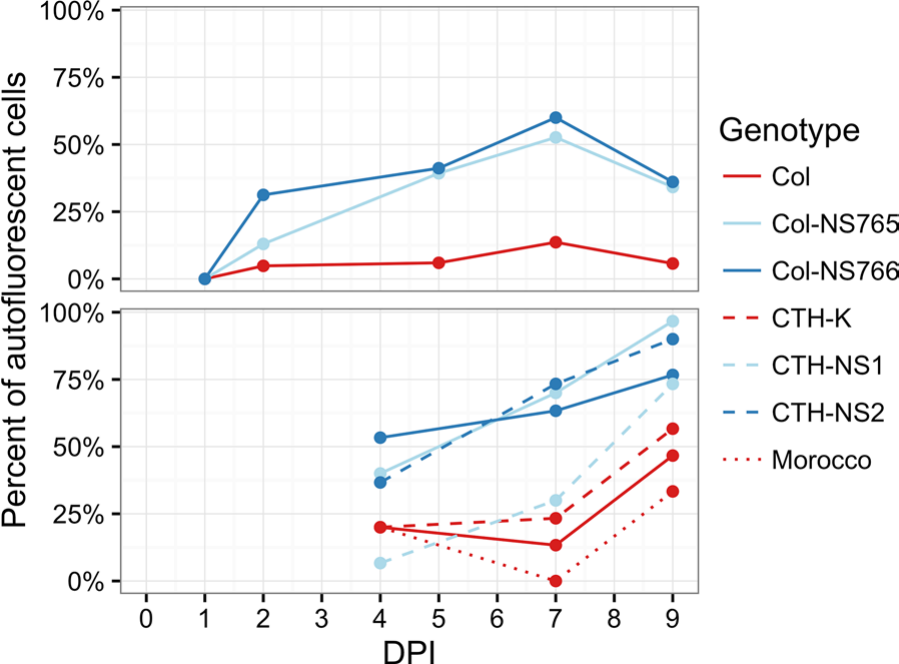
Autofluorescence. Percentage of infection sites with autofluorescent cells through time. *Panels* represent independent experiments for which (*Top*) all infection sites were analysed (mean = 60) and (*Bottom*) the first 30 infection sites were analysed, on the pooled first-leaf of two seedlings. Susceptible genotypes are represented in *red* and resistant genotypes in *blue*

**Fig. 4:**
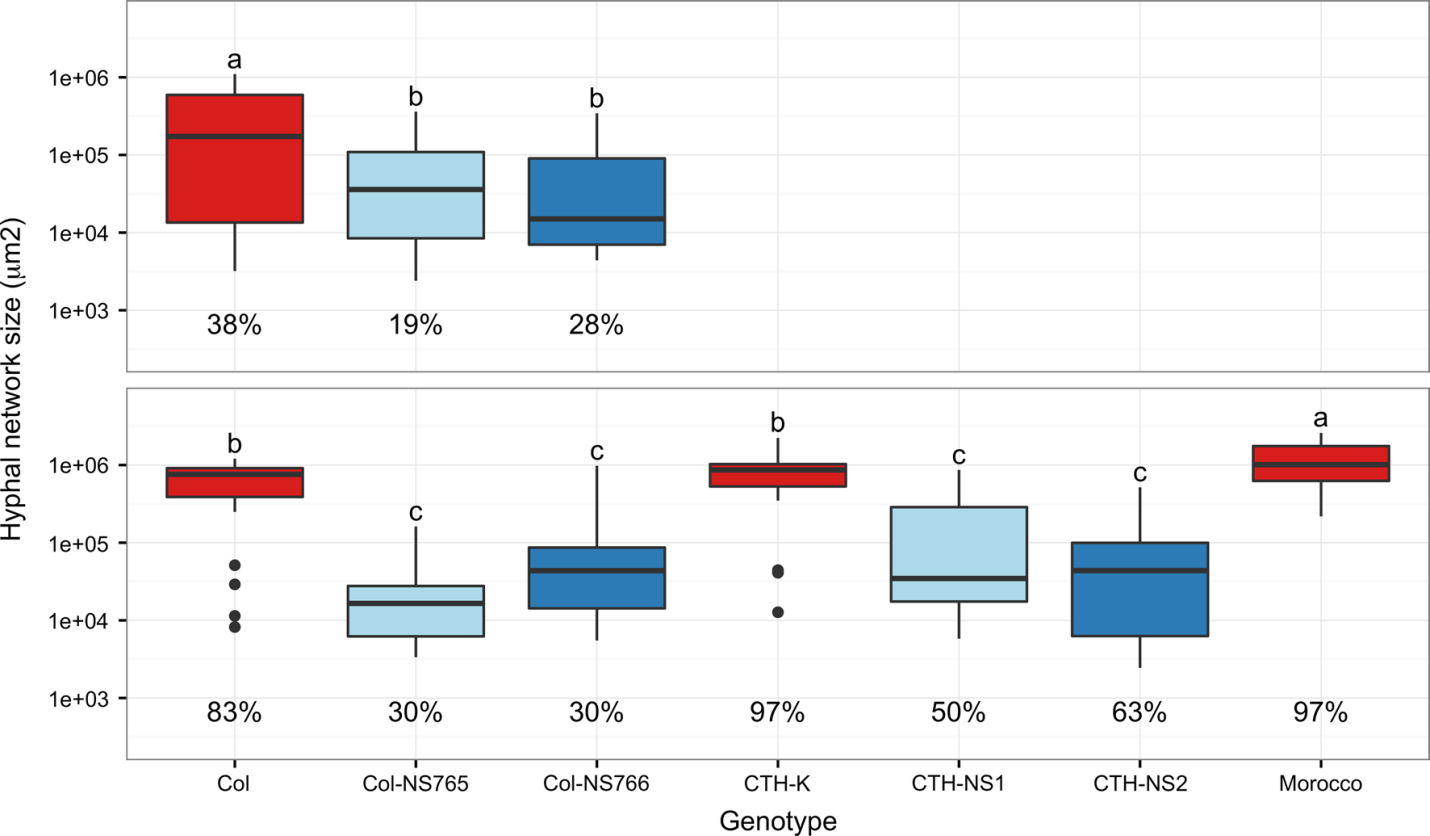
Hyphal network size. *Boxplot* representing the size distribution of hyphal networks at 9 DPI in each genotype. The median is represented by the *horizontal bold line*, lower and upper hinges correspond to the first and third quartiles, the whiskers extend from the hinges to the highest and lowest value within 1.5 of the inter-quartile range and points beyond the whiskers are considered outliers. The percentage of stem rusts with a developed hyphal network is indicated below the boxes and groups with *different letters* are significantly different (LSD, p value <0.05). *Panels* represent independent experiments for which (*Top*) 105, 79 and 61 infection sites were analysed in Col, Col-NS765 and Col-NS766, respectively, and (*Bottom*) the first 30 infection sites were analysed, on the pooled first-leaf of two seedlings. Susceptible genotypes are represented in *red* and resistant genotypes in *blue*

**Fig. 5:**
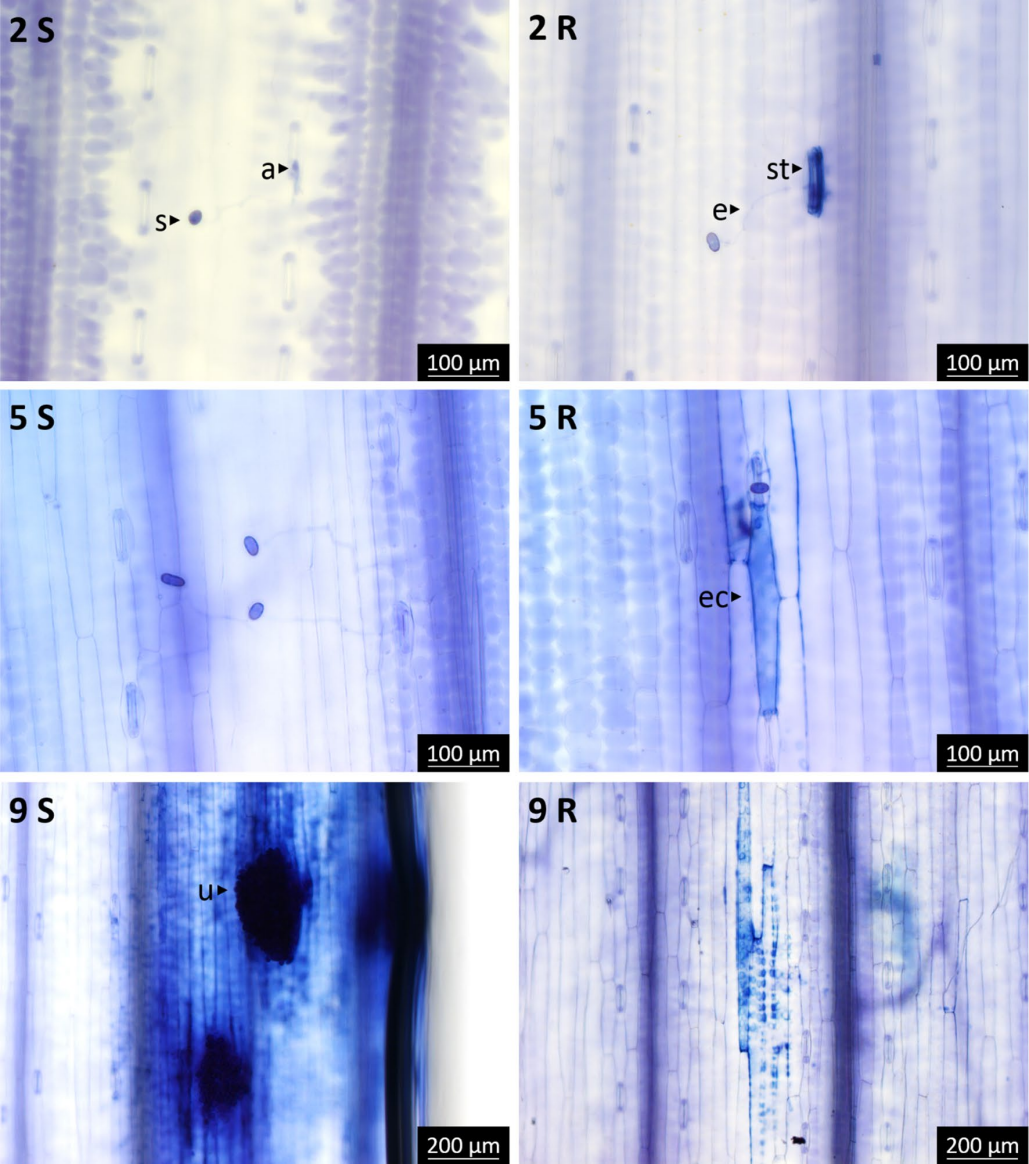
Trypan blue staining of responses to rust infection in resistant and susceptible genotypes. Time points are indicated by the corresponding numbers (DPI), and *S* and *R* represent the susceptible and resistant typical responses, respectively. *Arrows* indicate various rust and plant structures: urediniospore (s), elongation tube (e), appressorium (a), uredinia (u), stoma (st) and epidermal cell (ec)

**Fig. 6:**
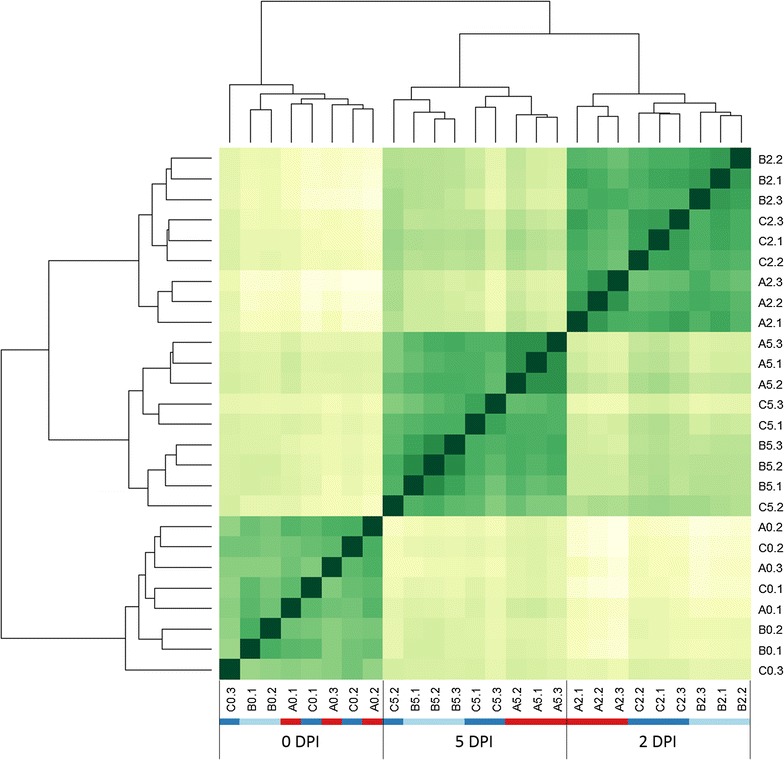
Hierarchical clustering. Dendrogram and heat map showing the Euclidian distances between the samples, based on normalized reads count for each Unigene; the darker the *color*, the smaller the distance between samples. *Letters* and *colored horizontal bars* represent genotypes (*A* and *red*: Col, *B* and *light blue*: Col-NS765, *C* and *dark blue*: Col-NS766). The *first numbers* represent time points (DPI) and the *last numbers* represent biological replicates

**Fig. 7:**
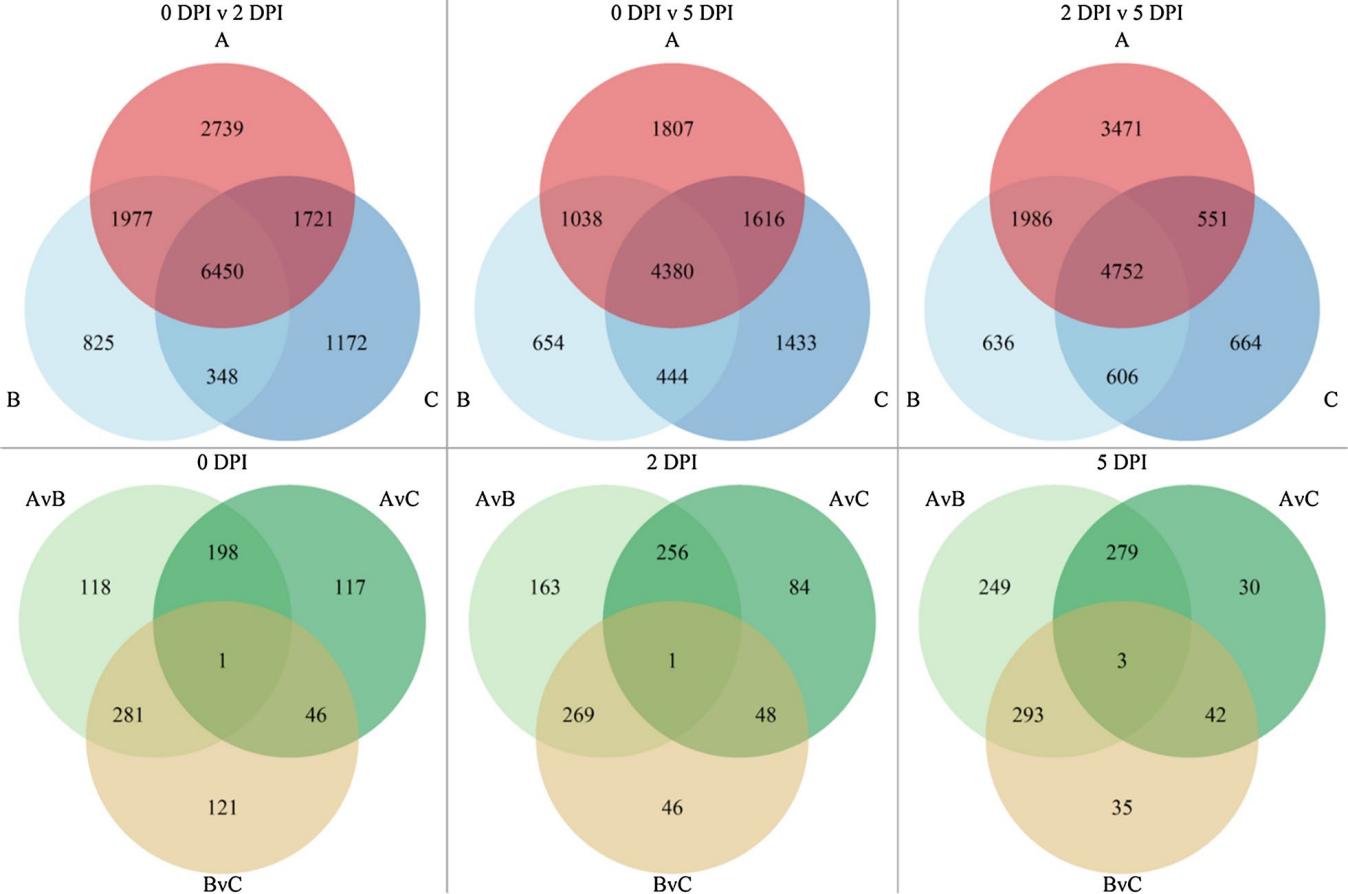
Venn diagrams of DE genes. (*Top*) Numbers of DE genes through time for each genotype. *Letters* represent genotypes: Col (*A*, *red*), Col-NS765 (*B*, *light blue*), Col-NS766 (*C*, *dark blue*). (*Bottom*) Numbers of DE genes between genotypes at each time point (DPI): Col versus Col-NS765 (*AvB*, *light green*), Col versus Col-NS766 (*AvC*, *dark green*) and Col-NS765 versus Col-NS766 (*BvC, brown*). The genes of greatest interest are those that were DE between Col and both Col-NS lines (i.e. intersection of AvB and AvC)

**Fig. 8:**
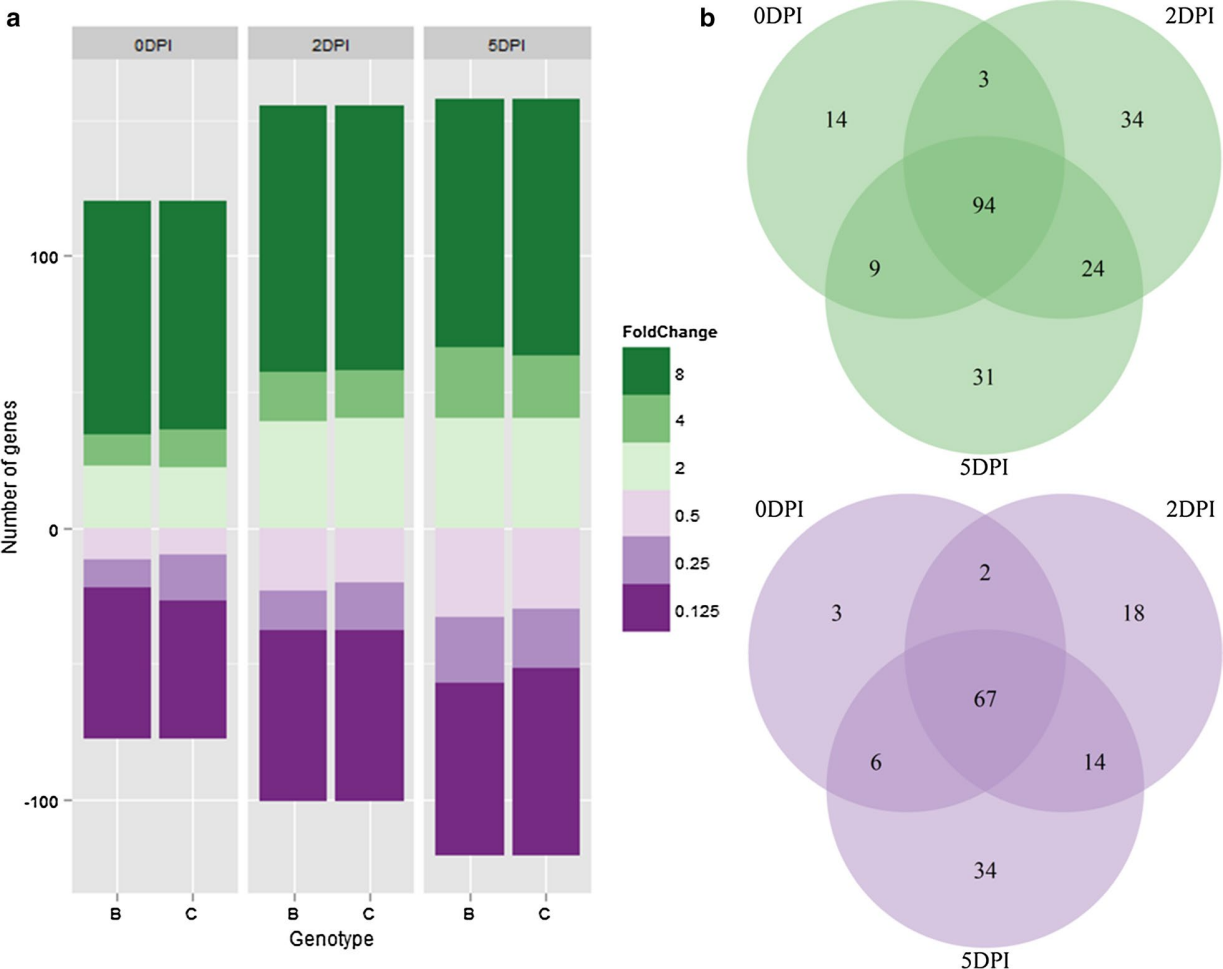
Genes of interest. **a** Number of genes of interest that were more (*up*, *green*) or less (*down*, *violet*) expressed in Col-NS765 and Col-NS766, compared to Col, at each time point (DPI). *Color* intensity represents minimum fold change. *Letters* represent genotypes: Col-NS765 (**B**) and Col-NS766 (**C**). **b** Venn diagrams of genes of interest that were more (*top*, *green*) or less (*bottom*, *violet*) expressed in both Col-NS lines compared to Col, at each time point (DPI)

Stem rust was visualized by staining with fluorescein isothiocyanate (FITC)-labelled wheat germ agglutinin (WGA), which binds to chitin present in fungal cell walls. Within 1 h of *Pgt* inoculation (0 days post inoculation—DPI), adhered urediniospores to the leaf surface were observed (Fig. 2-0). At 1 DPI (24 h after inoculation), most spores had germinated, but very few had developed an appressorium (Fig. 2-1). No difference between genotypes was observed at these time points. At 2 DPI, appressoria were formed over stomata as the pathogen started entering the leaf (Fig. 2-2). Some guard cells of the resistant lines showed blue autofluorescence under UV light, indicative of cell death (Fig. 2-2R2). This phenomenon was always associated with an infection site (i.e. the location where appressoria formed, typically at the stoma), and a penetration peg was often present. Although also observed in Col, autofluorescence of plant cells around stem rust infection sites was less frequent in the susceptible line than in the resistant ones (Fig. 2-2S2), and this applies to any time point from 2 DPI (Fig. 3). At 4 and 5 DPI, the rust colonies were well established inside the leaf of Col as many hyphae were observed, forming a small network (Fig. 2-5S). Haustoria were also visible. For the resistant lines, most fungi were still at the penetration stage and few hyphal networks and haustoria were observed. Epidermal cells adjacent to these infection sites were often autofluorescent under UV (Fig. 2-5R). Some mesophyll cells were also autofluorescent. At 7 DPI, hyphal networks spread further in Col (Fig. 2-7S), whereas their progression was limited in Col-NS765 and Col-NS766 (Fig. 2-7R). Finally, at 9 DPI, hyphal networks were large in Col, and urediniospores had begun to form (Fig. 2-9S). Many of the leaf cells adjacent to the large rust pustules showed blue autofluorescence. In the resistant genotypes, the relatively few fungal networks that progressed beyond the penetration stage were mainly smaller than in Col, and also showed autofluorescence of surrounding plant cells (Figs. 2-9R, 4). These results were in agreement with macroscopic observations at later time points, i.e. more and bigger uredinia in the susceptible genotypes (Fig. 1).

Observations in the susceptible CTH-K were comparable to those of Col, while the resistant lines, CTH-NS1 and CTH-NS2, were comparable to Col-NS765 and Col-NS766. At 9 DPI, more and highly extended hyphal networks were present in the susceptible lines than in the resistant ones (Fig. 4). Also, more rust infection sites were arrested at the penetration stage in lines with Col background than in lines with CTH background, reflecting the slightly higher susceptibility of the CTH background (Fig. 4).

#### Trypan blue staining

As well as dead plant cells, rust structures on the leaf surface (spores, elongation tubes and appressoria) were visible after trypan blue (TB) staining, when observed within 24 h of de-staining (Fig. 5). Observations were similar to those with WGA-FITC: urediniospores attached to the leaf surface at 0 DPI, germinated at 1 DPI and developed appressoria over stomata between 1 and 2 DPI. The first sign of plant cell death was observed at 2 DPI (Fig. 5-2R). Guard cells were sometimes TB stained in the resistant lines but almost never in Col. TB staining of leaf cells was associated with the presence of urediniospores or appressoria. At 5 DPI, epidermal cells adjacent to infection sites were often stained in the resistant genotypes (Fig. 5-5R). At 9 DPI, pustules with urediniospores, surrounded by TB stained epidermal cells, were observed in Col (Fig. 5-9S), but not in the resistant lines (Fig. 5-9R). At this stage, the staining in Col was probably due to the extensive damage caused by the rust development and the formation of uredinia breaking through the plant epidermis.

### Transcriptome analysis

#### Reads alignment

mRNA was isolated from samples consisting of the first leaf of two seedlings at 0, 2 and 5 DPI. Sequencing yielded 36 million reads on average per samples, and a total of 982 million reads for the 27 samples (3 genotypes × 3 time points × 3 biological replicates). Reads were aligned against the wheat Unigene database (http://www.ncbi.nlm.nih.gov/unigene), corresponding to 158 K transcripts. Alignment yield averaged at 65% of the total read set. Pearson’s correlations of read counts for each Unigene showed positive correlations between all samples (mean 90%), with best correlations between biological replicates (mean 98%). However, one replicate of Col-NS765 at 0 DPI was not as well correlated, even with the corresponding replicates (average 77%), and was removed from the analysis.

Unigenes were filtered to those with a minimum of 100 total reads across all samples, resulting in a total of 67,156 Unigenes expressed across samples. Of these Unigenes, more than half had <30 aligned reads on average for each sample and around 75% had <100 aligned reads per sample (Additional file 1: Figure S1). 95% of the Unigenes were represented by <30% of the total reads. This implied that most of the sequenced reads were associated with few genes that were highly expressed. As expected, these Unigenes corresponded to those associated with photosynthesis and leaf metabolism, for example RuBisCO activase, with 500 K reads (2.5%) on average per sample.

#### Differential expression analysis

Read counts for each gene in each sample were first normalised using the R DESeq package [13]. Hierarchical clustering of all samples revealed three main clusters, each representing a time point (Fig. 6). Genotypes were grouped into 3 sub-clusters at 2 and 5 DPI, but not at 0 DPI. In addition, Col-NS765 and Col-NS766 were closer to each other, at 2 DPI, than to Col. At 5 DPI, Col and Col-NS765 clusters remained distinct, whereas it was not as clear for Col-NS766. Differential expression analysis was performed using DESeq. Eighteen pairwise comparisons were explored, corresponding to two categories: (1) genotype comparisons at each time point and (2) time comparisons for each genotype.

As seen above, most of the Unigenes were represented by relatively few reads per sample. In addition, mRNA was isolated from whole leaves, whereas gene expression differences were likely to be concentrated in cells in contact with the rust pathogen, at least early after inoculation. To allow for these attenuating factors, the conditions for calling DE genes were not made too stringent, with a two-step selection, in order to maximize the number of candidate genes. Genes were at first considered differentially expressed (DE) with false discovery rate (FDR) ≤ 0.05 and absolute log_2_ fold change (FC) ≥ log_2_(2). On average, 8130 (12.1%) and 470 (0.7%) genes were DE between time points and between genotypes, respectively (Fig. 7). Once DE genes were determined for a particular comparison (e.g. 0 vs 2 DPI in Col), these same genes were re-evaluated in similar comparisons (e.g. 0 vs 2 DPI in Col-NS766), using less stringent thresholds [FDR ≤ 0.075 and |log_2_(FC)| ≥ log_2_(1.8)], which allowed detection of false negatives with respect to differential expression; for example, Ta.34224 was initially found to be DE between 0 and 2 DPI in Col-NS765 and Col-NS766 (FDR ≤ 0.05 and FC = 2.92 and 2.07, respectively), but not in Col (FDR ≤ 0.05 and FC = 1.97), but a closer analysis showed their expression patterns were very similar in all three genotypes.

#### Gene expression validation

In order to make sure that expression levels of non-pathogen-responsive genes were comparable between samples, read counts for 12 wheat reference genes, selected from a study by Paolacci et al. [14], were evaluated (Additional file 2: Reference Genes). None of these genes were found to be DE between genotypes, and only two (Ta.16204 and Ta.44405) were found to be DE between time points, being down-regulated at 5 DPI, compared to 0 DPI, in all genotypes. The uniformity and stability of the expression of these reference genes, between genotypes and through time, demonstrated no underlying bias in the data and the analysis was considered valid. In addition, eight genes were also checked by RT-qPCR and results were in agreement with the RNA-Seq (Additional file 2: RT-qPCR).

#### Genes of interest for the stem rust resistance

Compared to 0 DPI, more than 15,000 and 11,000 genes were found to be DE in at least one genotype at 2 and 5 DPI, respectively (Fig. 7, Top). Around 40% of them were regulated in the same way in all genotypes, while several thousand genes were DE across time points in only one genotype. More genes were DE in Col across time points than in Col-NS765 and Col-NS766. A total of 1502 genes were actually DE between genotypes. At any time point, more genes were DE between Col and Col-NS765 than between Col and Col-NS766 (Fig. 7, Bottom).

Genes that were DE at any time point between Col and both backcrossed independent resistant lines, Col-NS765 and Col-NS766 (hereafter referred to collectively as Col-NS), were selected as genes of interest (GOIs); as these genes were potentially involved in the resistance response mediated by the 7AL locus. A total of 353 genes were selected, of which 198, 256 and 279 were DE at 0, 2 and 5 DPI, respectively (Fig. 7, Bottom). More than half of the GOIs were DE by at least eightfold (Fig. 8a). Fold changes were usually similar between Col and either Col-NS765 or Col-NS766. More than 80% of the genes that were DE between Col and Col-NS at 0 DPI were also DE at 2 and 5 DPI (161 genes, Fig. 8b). Also, 23 and 11% were never expressed in Col or Col-NS, respectively.

Unigenes with a minimum of 100 reads aligned (67,156) were assigned to a wheat chromosome by aligning the Unigene reference sequences to the IWGSC wheat chromosome survey sequencing (CSS) database [15]. More than 99% of the Unigenes were unambiguously associated with a chromosome. The 67,156 Unigenes were found to be uniformly distributed across the genome (Additional file 3: Figure S2). However, among the GOIs (353) that were DE at 0 DPI (198), a disproportionate number were found to be located on chromosomes 6AL, 6AS and 7AL (Additional file 3: Figure S2), which carried 41 (20.7%), 18 (9.1%) and 16 (8.1%) genes, respectively.

The 353 GOIs were classified into categories (Table 1), depending on the time points at which they were DE between Col and Col-NS and their expression through time. 134 (38%) genes were categorized as constitutively DE, that is, always DE between Col and Col-NS, but never through time (Table 1 “Constitutive”). 85 (24%) and 78 (22%) genes were categorized as putatively involved in the early and late resistance response, respectively, with 25 genes being in common between both resistance responses (Table 1 “Early” and “Late”). Finally, 27 (8%) genes were putatively involved in the initiation of the resistance response, as they were located on chromosome group 7L and DE between Col and Col-NS at 0 DPI (Table 1 “Origin”). Genes on 7BL and 7DL were also considered, in case they corresponded to 7AL homoeologues that were missing in the Unigene or wheat genome databases.

**Table 1:** Classification of genes of interest

Category	Definition	Criteria	Total		**More expressed in**			
					Col		Col-NS	
			#	%	#	%	#	%
Constitutive	Genes constitutively expressed	Always DE between Col and Col-NS, and never DE through time in any genotype	134	38%	59	44%	75	56%
Early	Genes putatively involved in the early resistance response	DE at 2 DPI between Col and Col-NS, but not at 0 DPI unless DE at 2 DPI, compared to 0 DPI	85	24%	36	42%	49	58%
Late	Genes putatively involved in the late resistance response	DE at 5 DPI between Col and Col-NS, but not at 0 DPI unless DE at 5 DPI, compared to 0 DPI	78	22%	32	41%	46	59%
Pathogen	Genes putatively from the pathogen	Not expressed at 0 DPI in any genotype, highly up-regulated in Col at 5 DPI	4	1%	4	100%	0	0%
Miscellaneous	Remaining genes		77	22%	9	12%	18	23%
Origin	Genes that map at the origin of the resistance (i.e. possible R gene)	DE between both Col-NS and Col at 0 DPI, and on chromosome 7L	27	8%	7	26%	20	74%

Criteria used for categorizing the genes found DE between Col and both Col-NS765 and Col-NS766, and the number and percent of the 353 genes of interest in each category

Genes were annotated using the rice, *Arabidopsis* and NCBI Refseq databases. Among the GOIs, 153 (43%) were annotated, including 40 genes (11%) annotated as repetitive or transposable elements, and 200 (57%) were poorly annotated or not at all. All details concerning the GOIs can be found in Additional file 2.

#### Hormonal pathways

The expression of five *PR* genes (*PR1*-*5*), usually associated with the salicylic acid (SA) and jasmonic acid (JA) pathways, was investigated (Additional file 2: PR genes). They were all found to be up-regulated at 2 and 5 DPI in all genotypes, without significant differences between the susceptible and resistant lines. The expression of *PR1* was checked by RT-qPCR and results were similar, whereas in an independent mock-inoculation experiment, *PR1* was not found to be up-regulated in Col and Col-NS766 between 0 and 5 DPI (data not shown).

## Discussion

### Microscopic observations

Stem rusts enter a leaf by forming an appressorium over a stoma, after which a penetration peg enters the leaf though the stomatal opening, eventually invading, usually a mesophyll cell, and forming a haustorium [16]. In this study, the different steps of stem rust development *in planta* were observed, from the landing of urediniospores on the leaf surface, to the formation of new spores 9 days later. Various rust structures were detected, such as appressoria and haustoria. Results indicate a two-phase resistance response, preceding and following plant invasion by the pathogen, which ultimately led to fewer uredinium sites with fewer spores in the resistant lines (Fig. 1).

The pre-invasion, or early resistance response, was visible from 2 DPI, after the fungus had developed an appressorium and started entering the leaf, but before haustorium formation (Figs. 2, 5). The response was localized to the stomatal guard cells and adjacent epidermal cells at the site of penetration, which showed greater autofluorescence or TB coloration in the resistant lines than in the susceptible line. Both autofluorescence and TB coloration are indicative of dead plant cells [17, 18], suggesting that the resistance involves host cell death, predominantly in the epidermal cell layer. This is possibly due to HR, which is typical of ETI [8]. HR is reported to usually involve the death of host mesophyll cells, after the development of haustoria [19]; however, others have also observed pre-haustorial resistance responses in incompatible, ETI-mediated interactions. For example, a pre-haustorial response to stem rust was recently reported by Wang et al. [20], who observed that early responses to avirulent stem rust races in wheat carrying resistance genes *Sr5* and *Sr36* involved callose deposition in stomatal guard cells. Another notable example of a pre-haustorial response to wheat stem rust is the case of barley durable stem rust resistance gene *Rpg1*, which encodes a receptor-like kinase (RLK) that is autophosphorylated within minutes of inoculation with the avirulent pathogen [21, 22]. Rpg1 is believed to interact, presumably in the epidermis, with two avirulence proteins present in urediniospores, resulting in the initiation of HR well before haustoria formation. Thus, the stem rust resistance mediated by the 7AL rust resistance locus shows some similarity with the mechanism of action reported for barley *Rpg1*.

As detailed above, Col was moderately susceptible (IT 2^+^3) to stem rust race 313, while the two Col-NS lines were resistant (IT;1). Death of epidermal cells adjacent to rust invasion sites was also observed in Col, although these responses were less frequent than in Col-NS. Therefore, the resistance mechanism in Col-NS seemed to be an enhancement of a weak resistance present in Col. Interestingly, a resistance response similar to the one described here was microscopically observed in all three genotypes following inoculation with an avirulent race (data not shown). This suggests that the same resistance mechanism is effective in all genotypes, but it was not activated in Col against race 313, whereas it was in Col-NS through the specific recognition of the pathogen, indicative of an ETI response. Further studies will be needed in order to establish whether the enhanced resistance in Col-NS is derived from a basal PTI response or a weak and early ETI response present in Col.

The early resistance response correlated with fewer rust infection sites progressing beyond penetration in Col-NS compared to Col (Fig. 4). For those that successfully invaded the plant, hyphal networks and uredia were usually smaller than in Col (Figs. 1, 4). This might be due to the early resistance response, which delayed rust propagation, or to the establishment of a late resistance response. This resistance response might also involve HR, as suggested by the autofluorescence and TB staining of mesophyll cells and the leaf necrosis/chlorosis at a later stage. However, compared to the early resistance response, cell death was not common, and the propagation of fungi that evade the early resistance could be hindered by another resistance mechanism.

### Transcriptome analysis

RNA-Seq is a powerful technique for quickly obtaining an overview of the molecular changes during plant-pathogen interaction. Wheat transcriptome studies during rust infection often rely on other techniques (e.g. microarray) and deal with leaf and stripe rusts [23–30]. To the best of our knowledge, the current study is the first wheat whole-transcriptome analysis during *Pgt* infection.

mRNA from the susceptible Col and the two resistant lines Col-NS765 and Col-NS766 was isolated at three time points, based on the microscopic observations: (1) 0 DPI to determine gene expression differences between the genotypes before the initiation of the response to rust inoculation, (2) 2 DPI to investigate gene expression differences in the early resistance response during leaf penetration, and (3) 5 DPI to investigate gene expression differences in the late resistance response during fungal invasion.

Hierarchical clustering analysis of the similarity between samples revealed a grouping into three main clusters, each representing one time point after inoculation (Fig. 6). Thus, the transcriptome profiles of all genotypes, at a given time point, were more similar to each other than those of a single genotype through time. In other words, gene expression changes through time after rust inoculation were broadly similar for all three genotypes. However, the genotypes grouped into three sub-clusters at 2 and 5 DPI, but not at 0 DPI, with Col-NS765 and Col-NS766 being clearly closer to each other than to Col at 2 DPI. This indicates that responses to stem rust diverged between the sensitive and resistant genotypes by 2 DPI. At all three time points, there were higher numbers of DE genes between Col and Col-NS765 than between Col and Col-NS766 (Fig. 7), indicating that the transcriptome profile of Col was more similar to that of Col-NS766. A possible explanation for these results is that more of the CTH-NS genetic background remains in the backcrossed line Col-NS765 than in Col-NS766.

#### Genes of interest

The study was designed to identify gene expression differences relevant to the mechanism of resistance mediated by the 7AL stem rust resistance locus. The DE genes on which we focused were those that were DE between Col and both Col-NS765 and Col-NS766 backcrossed lines (total 353 GOIs). These genes were expected to include two categories: (a) genes derived from the original CTH-NS resistant backgrounds, and selected, along with the resistance phenotype, through five backcrossed generations, in both of the two independent Col-NS lines [11], and (b) genes in the Col genetic background that were regulated by genes in the first category.

Interpretation of the results of the analysis suffered from the limitation in annotation of wheat genes, with good annotations available for only 43% of the GOIs. Furthermore, several of the GOIs were actually found to be from rust pathogens despite being represented in the wheat Unigene database. These genes (“Pathogen” category in Additional file 2) were not expressed in any of the wheat genotypes at 0 DPI, but were expressed at higher levels at 2 and 5 DPI, and were more highly expressed in Col than in Col-NS, providing a measure of the greater proliferation of the pathogen in the more susceptible genotype.

As shown in Additional file 3: Figure S2, the 67 K Unigenes with >100 aligned reads were more or less evenly distributed across all wheat chromosomes, as indicated by alignment of the 67 K Unigene sequences with the wheat CSS database. The 353 GOIs were also roughly uniformly distributed through the genome, with two notable exceptions, chromosomes 7AL and 6A, each of which contained a disproportionately high number of the GOIs (Additional file 3: Figure S2 middle panel) Most of the GOIs assigned to these chromosomes were among the GOIs that were DE at 0 DPI, i.e. they were DE between the susceptible and resistant genotypes before any response to the pathogen was evident (Compare middle and bottom panels in Additional file 3: Figure S2). A likely explanation for this pre-existing difference is that they represent alleles inherited from the CTH-NS parent lines in the two crosses, and were co-selected with stem rust resistance through five backcrossed generations in the two independent Col-NS lines.

These findings are consistent with the mapping of a locus of major effect on the resistance to chromosome 7AL using a 9 K SNP genotyping assay and restriction site-associated DNA sequencing (RAD-Seq) [9]. The mapping study also revealed a high degree of polymorphism between the parental genotypes on chromosome 6A. Analysis with several molecular markers further indicated that Col-NS765 shares some regions on 6A with Col-NS766, but not with Col, consistent with these regions being inherited in the two independent backcrossed lines from the CTH-NS parents [9]. However, any association of markers on chromosome 6A with the resistance was masked by the segregation of the 7AL locus in this population. In the current study, the disproportionately large number of GOIs located on chromosome 6A also suggests the presence of genes affecting resistance on this chromosome. Further mapping using populations not segregating for the 7AL locus will be needed to clarify the role of chromosome 6A in the resistance.

Among the 59 GOIs detected on chromosome 6A that were DE between Col and Col-NS at 0 DPI (i.e. the genes that were most likely introgressed in the Col background), only 10 had useful annotations. Among them, two genes present may be involved in resistance. Ta.71325 encodes an ATP/ADP transporter similar to AATP1/ATNTT1, for which mutants in *Arabidopsis* and decreased activity in potato were found to increase resistance to several pathogens [31, 32]. However, expression of this gene in this study was initially higher in Col-NS than in Col but then increased in this last to a comparable level. Ta.72561, on the other hand, was always more expressed in Col. It encodes a Myosin-like protein with homology to Myosins XI, which were found in *Arabidopsis* to be regulators of early plant antifungal immunity at the penetration site [33].

##### GOIs on 7AL

As the major locus conferring resistance was mapped to chromosome 7AL, GOIs located on this chromosome represented the best resistance gene candidates among the differentially expressed transcripts. Twenty of the 353 GOIs mapped to chromosome 7AL (Additional file 2: All on chr 7L). A further 20 GOIs mapped to chromosomes 7BL and 7DL, representing possible homeologues of genes that may not be represented in the CSS database for 7AL. Of the 20 GOIs on 7AL, 8 had annotations, of which 4 encoded putative cysteine-rich receptor-like kinases (CRKs). One CRK located on 7DL (Ta.90847) encoded a protein with 97% identity to CRK Ta.90783 on 7AL, the former therefore representing a possible D homoeologue of the latter. Ta.109657 on 7BL encoded a putative CRK protein with 82% identity to putative CRKs encoded by Ta 95070 and Ta.75386, both on 7AL. At this level of similarity, the 7BL gene probably represents a member of a family containing all three genes rather than being a homoeologue of either 7AL gene. However, the Ta.109657 Unigene sequence was short, encoding only 95 amino acids compared to the approximately 350 amino acids of the other proteins, so any comparison involving this sequence would not give an accurate representation of the similarity between full-length proteins.

Receptor-like kinases (RLKs) are signal transducers generally containing an N-terminal, extra-cellular receptor domain, a trans-membrane domain, and an intra-cellular protein kinase domain at the C-terminus. The large protein family is categorised according to the motifs recognisable in the predicted extra-cellular domains; notably, leucine-rich repeats characterise the nucleotide-binding leucine-rich RLKs (NLRs) involved in recognition of specific pathogens at the cell surface, and subsequent activation of ETI in plants [34]. CRKs comprise another large subfamily of RLKs, members of which are characterized by the conserved C-X8-C-X2-C motif (DUF26) in their extra-cellular domains [35]. However, using a variety of web-based tools, neither the DUF26 motif, nor trans-membrane domains, were recognisable in the predicted proteins encoded by the 7AL CRK Unigene sequences, indicating that the predicted proteins are cytoplasmic, and do not in fact belong to the CRK subfamily. Rather, they should be considered as receptor-like cytoplasmic kinases (RLCKs).

RLCKs make up a large sub-family of RLKs. They consist of a predicted protein kinase domain, with no targeting signals, indicating that they are cytoplasmic. RLCKs have been demonstrated to function in the cascade of plant defence signalling by interacting with pattern recognition receptors and associated proteins at the inner surface of the plasma membrane, subsequently transducing the signal perceived at the cell surface to induce PTI defence responses. Furthermore, it has been demonstrated that plant RLCKs are targets of pathogen avirulence proteins, which can attenuate plant PTI by modifying a target RLCK [36]. The wheat 7AL putative RLCKs have no close homologues amongst well-characterised kinases, but their kinase domains show similarity to the kinase domains of CRKs (30–40% amino acid identity), hence the annotations. Notably, the 7AL putative RLCKs also show similarity to the two kinase domains of barley Rpg1 (for example 40 and 37% identity between the single kinase domain of the Ta.90783 predicted protein and the two kinase domains of Rpg1). As noted above, the microscopic observations reported here indicate some similarities in the mechanisms of action of the wheat 7AL resistance locus and the well-characterised stem rust resistance gene *Rpg1* in *Hordeum vulgare* [22]. Thus the 7AL RLCKs exhibited characteristics of proteins that could have a role in plant defence. Remarkably, comparison of the 7AL contigs containing the GOIs with the 7AL contigs containing SNPs mapped to the 7AL resistance locus in previous work, showed that two of the DE GOIs, both encoding putative RLCKs, mapped at or near the 7AL resistance locus. RLCK Ta.90783 was located on 7AL contig 4487195, which co-segregated with the cluster of markers most closely linked with the stem rust resistance, while Ta.104804 was located on 7AL contig 4519254, which was separated by 11 recombinants from the cluster of co-segregating markers [9]. Ta.90783 therefore represents a strong candidate for further investigation.

##### Early resistance response

Eighty-five genes were found to be DE between Col and Col-NS at 2 DPI, and thus putatively involved in the early resistance response, of which 36 and 49 were more expressed in Col and Col-NS, respectively. Many presented similar expression patterns in all genotypes, which could suggest that they are not responsible for differentiation between resistance and susceptibility. Nevertheless, the differential expression of these genes could have a quantitative effect on the resistance. Twenty-eight had annotations other than repetitive/transposable elements. Interestingly, some genes that could be involved in defence, such as Ta.75060 (disease resistance) and Ta.13785 (xylanase inhibitor) [37] were more expressed in Col. These genes may be part of the weak defence response in Col, but the lower expression in Col-NS suggests they do not contribute, to any large extent, to the 7AL resistance response. Conversely, the down-regulation in Col-NS of Ta.10772, which encoded a nodulin MtN2-like, could hinder the pathogen progression [38]. Concerning the genes that were more expressed in Col-NS, three of them (Ta.90783, Ta.111478 and Ta.101645) were annotated as CRKs. They were also more expressed at 5 DPI. These genes, as well as Ta.45840 (calmodulin-binding protein) and Ta.1164 (serine/threonine-protein kinase), could be involved in signal transduction.

##### Late resistance response

Seventy-eight genes were found to be putatively involved in the late resistance response, of which 32 and 46 were more expressed in Col and Col-NS, respectively. Thirty-three had annotations other than repetitive/transposable elements. Of those more expressed in Col-NS, many were of particular interest; Ta.66505, Ta.87731 and Ta.88423 (disease resistance proteins), Ta.101645, Ta.102188, Ta.103989, Ta.111478 and Ta.90783 (CRKs), Ta.72300 and Ta.87792 (RLKs), Ta.78049 (ABC transporter [39]) and Ta.90050 (UDP-glycosyltransferase [40]) could all be part of the defence response.

#### Hormonal pathways

The SA and JA pathways are well known for being involved in plant immunity and are often described as antagonists in the resistance against biotrophic and necrotrophic pathogens [41, 42]. SA- and JA-signalling promotes the activation of the systemic acquired resistance (SAR) and the production of pathogenesis-related (PR) proteins. In this study, the up-regulation at 2 and 5 DPI of five *PR* genes in all genotypes strongly suggests that the SA and/or JA pathways have been activated in all three genotypes following rust infection, but the defence response in Col-NS is independent of or in parallel with these pathways.

## Conclusion

In this study, we investigated the resistance conferred by the newly-discovered wheat 7AL stem rust resistance locus. The data presented here suggest that the resistance conferred by the 7AL locus involves the death of stomatal guard cells and adjacent epidermal cells during the penetration stage. The combination of microscopy, transcriptomics, and mapping point to the mechanism of resistance in Col-NS being an enhanced form of a weak resistance present in Col that potentially involves RLCKs encoded at the 7AL locus. It is possible that these kinases interact to enhance either a basal PTI mechanism or weak ETI response. The great number of transcript sequences generated here provides a good basis for future research on *Pgt*-wheat interaction.

## Methods

### Plant material

All Canthatch (CTH-K, CTH-NS1 and CTH-NS2) and Columbus (Col, Col-NS765 and Col-NS766) lines/mutants were provided by Kerber [11], Agriculture Canada Research Station in Winnipeg, Canada. CTH-K is a single seed derived and purified selection of CTH descent from which the NS EMS mutants originated. CTH-NS1 and CTH-NS2 are two independent EMS mutants, in which the 7D-Sup has been inactivated [11]. Col-NS765 and Col-NS766 are near-isogenic lines, derived from independent backcrosses (BC_5_F_4_) of each of the two CTH-NS mutants with the recurrent parent Col. Both CTH and Col are closely related to Thatcher.

### Stem rust inoculation

Resistance to stem rust was evaluated with wheat stem rust *Pgt* culture no. 313 (pathotype 34-1,2,3,5,6,7 according to Australian nomenclature) from the Plant Breeding Institute, University of Sydney, Australia. This race is avirulent on *Sr9e* and *Sr21*, but virulent on *Sr5*, *Sr6*, *Sr7b*, *Sr8a*, *Sr9b*, *Sr9* *g*, *Sr11*, *Sr15* and *Sr17*. Seedlings were grown in a microclimate room (22/20 °C day/night) until the two-leaf stage (approximately 2 weeks). Inoculation was undertaken by spraying the plants with urediniospores suspended in light mineral oil. Plants were dried for 30 min and incubated for 2 days in incubation cabinets (22/20 °C day/night, high humidity). Finally, plants were transferred to a microclimate room, at a lower temperature (18/17 °C day/night), until sampling/scoring. Seedlings were scored for stem rust resistance between 11 and 15 DPI, using the stem rust scoring scale [43, 44].

### Microscopic observations

For each genotype, the first leaf of two seedlings from different pots were pooled at several DPI and stained for microscopic observations, using the Zeiss Axio Imager. M1 microscope. The development of stem rust *in planta* was observed using WGA-FITC, which binds specifically to N-acetyl-glucosamine (i.e. chitin) and allows the observation of fungal structures as green fluorescence. WGA-FITC staining was done following a modified protocol described by Ayliffe et al. [45]. Briefly, leaf tissue was immersed in 1 M KOH and incubated at 37 °C for 2 days; KOH solution was replaced during incubation. Tissue was then neutralized in 50 mM Tris, pH 7.0, and stained with WGA-FITC at 20 µg/ml (Sigma-Aldrich, St. Louis). Fungal structures and autofluorescence were observed under blue (excitation 470–440 nm, emission 525–550 nm) and UV (excitation 365 nm, emission ≥ 420 nm) light, respectively. This experiment was performed twice: at 0, 1, 2, 5, 7 and 9 DPI on genotypes with Columbus background for the first one, and at 4, 7 and 9 DPI on all genotypes for the second one. For each genotype, hyphal network size at 9 DPI was quantified by measuring the area covered by hyphae, using the AxioVs40 software (v4.8.20). In the first experiment, all infection sites were analysed, i.e. 105, 79 and 61 infection sites for Col, Col-NS765 and Col-NS766, respectively, whereas in the second experiment, the first 30 infection sites observed were analysed. Trypan blue staining was done once on genotypes with the Columbus background, following the method described by Desmond et al. [46].

### Transcriptome analysis

For each sample, the first leaf of two seedlings from different pots were pooled and quickly frozen in liquid nitrogen. Total RNA was extracted using the RNeasy Plant Mini Extraction Kit (Qiagen) and RNA integrity was checked on 1.4% agarose gel and on the Agilent 2100 Bioanalyzer. Following poly(A) isolation step performed by the sequencing company (Australian Genome Research Facility), mRNA samples were sequenced using Illumina HiSeq 2000 for 100 bp single-end and an expected yield of 30 million reads. Reads were aligned against the wheat (*Triticum aestivum*) Unigene database build #62 (http://www.ncbi.nlm.nih.gov/unigene), comprising 158,028 transcripts, using the BioKanga software (http://code.google.com/p/biokanga). Alignment allowed a mismatch rate of up to 10% of the length of the read. Only reads with a unique best hit were selected for differential expression analysis. Unigenes were filtered to those with a sum of counts greater than, or equal to, 100 across samples. Read counts were normalized and tested for differential expression, using the R DESeq package v1.12.0 with default parameters [13]. Hierarchical clustering was done using the Euclidian distances on the normalized reads count following DESeq variance stabilizing transformation. Eighteen pairwise comparisons were performed (between genotypes at each time point and between time points for each genotype). Genes were considered DE for FDR ≤ 0.05 and |log_2_(FC)| ≥ log_2_(2), and for FDR ≤ 0.075 and |log_2_(FC)| ≥ log_2_(1.8) if already found DE in similar comparisons. Genes were considered expressed if more than 0.15 read per kilo base per million (RPKM) was detected. Chromosomal location of each Unigene was determined using BLASTN (best hit, evalue ≤ 10^5^) against the flow-sorted IWGSC chromosome survey sequencing contigs of cultivar Chinese Spring [15]. Unigenes putatively involved in hormonal pathways (PR genes) were recovered using tBLASTN from wheat protein sequences. RT-qPCR checks were performed using the Applied Biosystems 7900HT Fast Real-Time PCR System and the relative expression level was calculated by comparing the target gene with the reference Unigene Ta.2291 (ADP-ribosylation factor) using the 2^−ΔCq^ formula.

## Additional files

**Additional file 1.** Histogram of read count. Bars represent the number of Unigene for a particular number of reads, and blue circles represent the additive percent of Unigene. Blue line represents the additive percent of reads.

**Additional file 2.** Supporting data. Details concerning the 353 GOIs, reference and PR genes, and RT-qPCR results.

**Additional file 3.** Genomic distribution of Unigenes. Top: the percent of total expressed Unigenes (67,156) on each chromosome. Note: chromosome 3B includes both 3BL and 3BS. Middle: the number of the 353 genes of interest (GOIs) on each chromosome, expressed as a percent of the total number of expressed Unigenes assigned to that chromosome (percent of Unigenes per chromosome). Bottom: the number of the 198 GOIs that were DE at 0DPI on each chromosome expressed as a percent of the total number of expressed Unigenes assigned to each chromosome.

## Abbreviations

CSS: IWGSC wheat chromosome survey sequencing
Col: Columbus
CTH: Canthatch
DE: differentially expressed
DPI: days post-inoculation
ETI: effector triggered immunity
FC: fold change
FDR: false discovery rate
GOIs: genes of interest
HR: hypersensitive response
IT: infection type
NS: non-suppressor
*Pgt*: *Puccinia graminis* f. sp. *tritici*
PTI: pattern triggered immunity
R-genes: resistance genes
SAR: systemic acquired resistance
TB: trypan blue
WGA-FITC: fluorescein isothiocyanate-labeled wheat germ agglutinin
