# Supplementary material for: Cellular and molecular characterization of a stem rust resistance locus on wheat chromosome 7AL
Source: BMC Res Notes. 2016 Dec 7;9:502. doi: 10.1186/s13104-016-2320-z (PMC5143453; doi:10.1186/s13104-016-2320-z)
Supplement: Supplementary file 1 — Additional file 1. Histogram of read count. Bars represent the number of Unigene for a particular number of reads, and blue circles represent the additive percent of Unigene. Blue line represents the additive percent of reads. [file 13104_2016_2320_MOESM1_ESM.pdf]

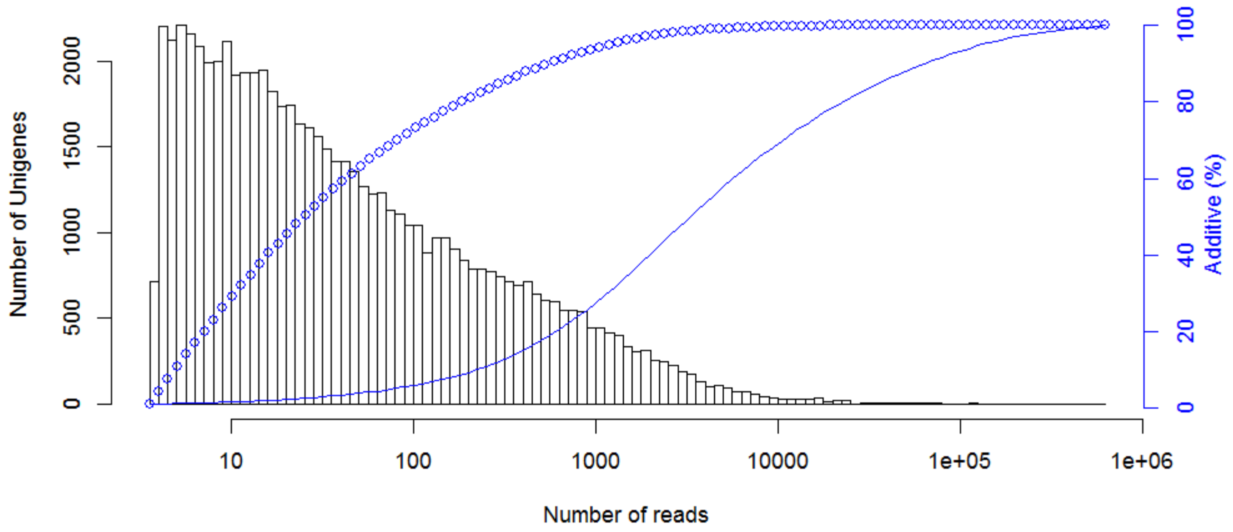

Histogram of read count. Bars represent the number of Unigene for a particular number of reads, and blue circles represent the additive percent of Unigene. Blue line represents the additive percent of reads.
