# Supplementary material for: Cellular and molecular characterization of a stem rust resistance locus on wheat chromosome 7AL
Source: BMC Res Notes. 2016 Dec 7;9:502. doi: 10.1186/s13104-016-2320-z (PMC5143453; doi:10.1186/s13104-016-2320-z)
Supplement: Supplementary file 3 — Additional file 3. Genomic distribution of Unigenes. Top: the percent of total expressed Unigenes (67,156) on each chromosome. Note: chromosome 3B includes both 3BL and 3BS. Middle: the number of the 353 genes of interest (GOIs) on each chromosome, expressed as a percent of the total number of expressed Unigenes assigned to that chromosome (percent of Unigenes per chromosome). Bottom: the number of the 198 GOIs that were DE at 0DPI on each chromosome expressed as a percent of the total number of expressed Unigenes assigned to each chromosome. [file 13104_2016_2320_MOESM3_ESM.pdf]

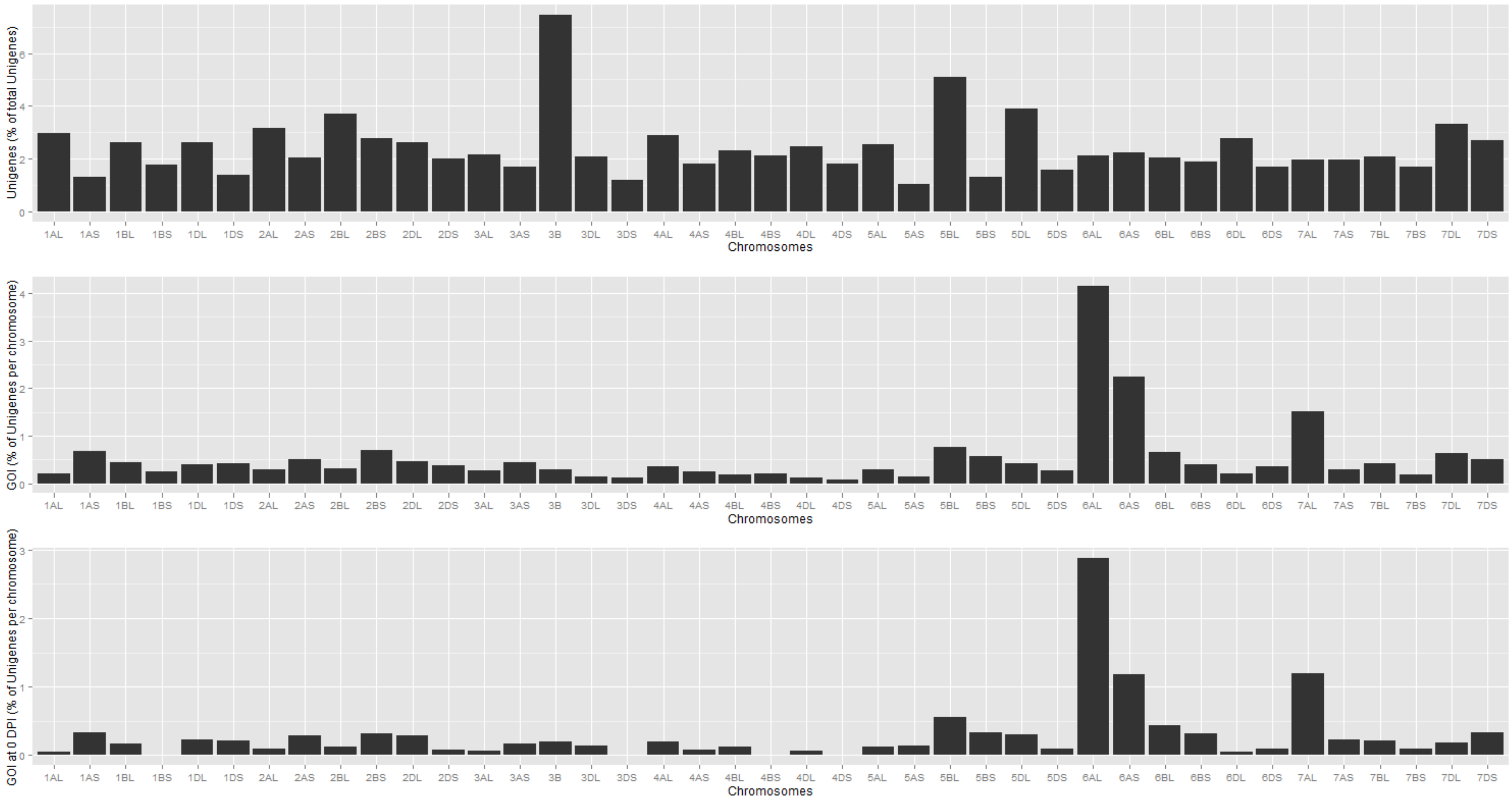

Genomic distribution of Unigenes. Top: the percent of total expressed Unigenes (67,156) on each chromosome. Note: chromosome 3B includes both 3BL and 3BS. Middle: the number of the 353 genes of interest (GOIs) on each chromosome, expressed as a percent of the total number of expressed Unigenes assigned to that chromosome (percent of Unigenes per chromosome). Bottom: the number of the 198 GOIs that were DE at 0DPI on each chromosome expressed as a percent of the total number of expressed Unigenes assigned to each chromosome.
